# How does the learning environment support vocational student learning of domain-general competencies?

**DOI:** 10.1007/s12186-023-09318-x

**Published:** 2023-04-06

**Authors:** Sami Löfgren, Liisa Ilomäki, Jari Lipsanen, Auli Toom

**Affiliations:** 1 Faculty of Educational Sciences, University of Helsinki, P.O. BOX 9, FI-00014 Helsinki, Finland; 2 Technology in Education Research Group, Faculty of Educational Sciences, University of Helsinki, P.O. BOX 9, FI-00014 Helsinki, Finland; 3 Department of Psychology and Logopedics, Faculty of Medicine, University of Helsinki, P.O. BOX 21, FI-00014 Helsinki, Finland; 4 Centre for University Teaching and Learning, Faculty of Educational Sciences, University of Helsinki, P.O. BOX 9, FI-00014 Helsinki, Finland

**Keywords:** Vocational education & training, Competence, Learning environment, Vocational students

## Abstract

Studies and policy reports worldwide argue that a modern employee must possess domain-general competencies to become employed. However, competency studies within upper-secondary initial vocational education and training are scarce. Therefore, this study aimed to scrutinise this topic and examined how the experienced learning environment contributes to student learning of competencies. Study participants were students in automotive engineering, mechanical and metal engineering, electrical and automation engineering and building service technology. The data were collected with an online questionnaire and analysed statistically using structural equation modelling. The research findings indicate firstly that eight competency domains could be recognised: work organisation, cooperation ability, professional attitude, problem solving, willingness to learn, active listening, empathy and assertiveness. Secondly, students’ experienced learning environment was characterised by social support and recognition provided by educators, equal treatment between students and a positive climate for learning. Thirdly, the quality of the experienced learning environment contributed to learning of competencies. The research findings enhance the scientific and societal discussion about vocational graduate competencies and to what extent the experienced learning environment contributes to the learning of competencies.

**Supplementary Information:**

The online version contains supplementary material available at 10.1007/s12186-023-09318-x.

## Introduction

The constantly changing world of work, evolving technology and political aspirations for labour mobility challenge vocational education systems worldwide (Mulder & Winterton, 2017; OECD, 2020). According to OECD (2020, 245) predictions, 14% of all jobs may become automated in the near future while 32% of jobs will be altered profoundly; especially jobs based on an upper-secondary initial vocational education and training (IVET) diploma risk becoming obsolete.

To counteract the deterioration of vocational schooling, vocational education systems should prepare students for life-long learning and provide graduates with eligibility for tertiary-level studies (OECD, 2020). Basically, an initial vocational education provides students with a formal, trade-specific qualification referring to domain-specific competencies (Gekara & Snell, 2018; Nägele & Stalder, 2017; OECD, 2020). Still, as the youth cannot acquire broad professional experience during their studies, they cannot distinguish themselves with domain-specific competencies in recruitment (Löfgren et al., 2020). Therefore, students must also develop domain-general competencies, which signify integrated sets of knowledge, skills and attitudes assisting individuals to adapt to the new and unknown (e.g., Blömeke et al., 2015; Gekara & Snell, 2018; OECD, 2020). For example, students can outperform other job applicants with their advanced social skills and willingness to learn (Nägele & Stalder, 2017). The IVET student perspective on their learning of domain-general competencies has not been often studied (Billett, 2014), especially employing quantitative self-report instruments (Panadero et al., 2018). In this study, we want to discover which domain-general competencies Finnish upper-secondary IVET students in technical trades perceive to learn during their studies.

Competencies do not develop in a vacuum but require an advanced learning environment. In fact, when the vocational learning environment is well established, it counterbalances individual shortcomings. For example, the learning environment may support students with low motivation to proceed with their studies (Virtanen et al., 2014). Many earlier studies have examined the relation of the experienced learning environment and students’ vocational development, especially during workplace learning (e.g., Böhn & Deutscher, 2021; Mikkonen et al., 2017); still, school contexts have not been studied as often (Lüthi et al., 2021). There is also a need to examine in more detail how students perceive their development in relation to educator behaviour (Ryökkynen et al., 2019) or pedagogical ethos (Forster-Heinzer, 2020). Further, many previous studies have underlined how the cultures, rationales and goals of workplace and school-based learning environments differ from each other (e.g., Aarkrog, 2005; Rintala & Nokelainen, 2020). While undeniable differences exist, most vocational students tend to perceive vocational schools and workplaces as relatively aligned learning environments in terms of educator supportiveness and learning climate (Lüthi et al., 2021). In fact, technical-trade vocational teachers and workplace supervisors (i.e., vocational educators) tend to resemble each other in their pedagogical attitude, dedication and practices (Goodson & Ümarik, 2019; Löfgren et al., 2022; Nylund & Gudmundson, 2017).

According to earlier studies (e.g., Forster-Heinzer, 2020; Lüthi et al., 2021; Soini et al., 2015; Toom et al., 2017), educators of a high-quality learning environment provide students with social support, equal treatment, a positive climate for learning and recognition of students’ endeavours. These features not only improve students’ motivation but also further their well-being, academic success, self-directed learning, identification with their profession and general vocational development (Forster-Heinzer, 2020; Jossberger et al., 2010; Ryökkynen et al., 2019). In this study, we want to test whether a supportive, equal, constructive and encouraging learning environment established by technical-trade vocational educators could contribute to student learning of competencies. Through this dual view of competency acquisition and learning environment experiences, we aspire to broaden the scientific and societal discussion on upper-secondary IVET students’ competency expectations and the preconditions for successful competency development.

## Theoretical background

### Competence as a multi-faceted concept

Competence as a concept reflects both one’s knowledge, ability for skilful performance and ability to adapt and act; thus, ‘competence’ is an emblem for one’s overall capability (Blömeke et al., 2015; Braun et al., 2012; Mulder, 2014; Mulder & Winterton, 2017; Toom, 2017). This perspective might be too broad to describe what one can do in each situation. In fact, competence can be divided into sub-clusters of knowledge, skills and attitudes, designated as ‘competencies’ (or ‘competency’ in singular; Mulder & Winterton, 2017). *Domain-specific competencies* equip one with trade-specific knowledge, skills and attitudes to perform and develop in certain job tasks (Gekara & Snell, 2018), for instance electrical assembly. *Domain-general competencies*, in turn, cannot be limited to specific trades but broadly prepare the worker for the unknown (Nägele & Stalder, 2017). Examples of these are professional attitude, communication and willingness to learn (e.g., Jossberger et al., 2010; Kyndt et al., 2014). Besides remaining employable, individuals with competencies can have better access to a good life and further education (Gekara & Snell, 2018; Mulder, 2014, 2019).

Despite some agreement about the definition of the concept of competence and its contents, these still constitute a long-lasting topic of academic discussion (cf. Blömeke et al., 2015; Mulder, 2014; Nägele & Stalder, 2017). For example, scholars studying vocational education have used such other concepts as occupational knowledge, capability, capacity, expertise and talent to describe competence; however, they all seem to cover relatively similar phenomena of human potential (Mulder, 2019).

For another, a fundamental idea behind the concept of competence (and its relatives) is that it benefits individual performance; however, scholars debate whether one’s competence should be seen as an inseparable, holistic entity or could it be analysed through its antecedents, in other words, as an array of single competencies (Blömeke et al., 2015; Mulder, 2014). There are three general perspectives to this dilemma within the competence research (Mulder, 2014). First, those scholars who argue for behaviouristic functionalism think that it is possible to analyse and define even minuscule domain-specific competencies; therefore, vocational teachers should concentrate on teaching these detailed skills to the students (Mulder, 2014). Second, those who support integrated occupationalism mix analytic and holistic views and highlight that students also need domain-general competencies; however, they tend to approach students’ competency needs from the workplace perspective (Mulder, 2014). Third, scholars who argue for situated professionalism take the utmost holistic stance. They consider that it’s the community of practice, which enables and acknowledges the development of one’s competence; however, this perspective hardly explains the actual curricular contents (Lave & Wenger, 1991; Mulder, 2014).

In practice, scholars or IVET systems do not categorically opt for a single perspective to competence (Blömeke et al., 2015; Mulder, 2014). First, vocational education aims to educate a skilful workforce to satisfy prevailing labour market competence demands (Gekara & Snell, 2018; Nägele & Stalder, 2017; OECD, 2020). Thus, even teenage IVET graduates should be able to conduct some basic professional tasks in their trades, often by hand (Löfgren et al., 2022; Mulder, 2014; Nägele & Stalder, 2017). Second, as many graduates lack experience, they cannot stand out from other job applicants with their basic-level domain-specific competencies; thus, they should rather distinguish themselves with domain-general competencies (Löfgren et al., 2020; Mulder, 2014). Third, workplace learning is an outstanding forum to develop one’s competencies and aspire for a future employment; therefore, many modern IVET systems combine theoretically driven school-based instruction with practically oriented workplace-based vocational training (Aarkrog, 2005; Mulder, 2014).

Yet another topic of discussion that relates to the previous one about competence and performance is competence assessment. In detail, such domain-specific competencies as usage of different tools or assembly techniques are relatively easy to define, select, articulate in the curricula and assess; domain-general competencies are the opposite as they are vaguer by nature and lack established assessment measures (cf. Kyndt et al., 2014).

There are multiple international policy-driven attempts to select and define the most important (domain-general) competencies, for instance the DeSeCo and PIIAC projects of the OECD (Braun et al., 2012; OECD, 2020). When frameworks are created, different political, economic, labour-market and educational stakeholders are consulted; thus, the result is an aligned ‘trade-off’ between different viewpoints (Mulder, 2014, 2019). Therefore, international frameworks tend to be too overarching to yield information on the local, trade-specific competency needs, or too abstract for adolescent IVET students (Kyndt et al., 2014; Mulder & Winterton, 2017). In fact, the contents of different competencies must be described concretely enough to ensure that they are attainable; ambiguous expectations only cause stress and anxiety for students (Atkins, 2013; Gekara & Snell, 2018).

### Vocational student learning of domain-general competencies

Instead of all-encompassing frameworks, few studies have highlighted on the grass-roots level which competencies local employers and vocational teachers expect of graduating vocational students. These studies suggest that graduates profit at least from having professional attitudes, problem solving and communication skills and a willingness to learn (Kyndt et al., 2014; Löfgren et al., 2020; Pylväs et al., 2018; Vähäsantanen & Hämäläinen, 2018). Finnish employers and teachers also consider IVET graduates’ levels of competencies to vary or even be unsatisfactory (Löfgren et al., 2020, 2022). However, students themselves have not often been consulted (Billett, 2014).

Panadero, Garcia and Faile (2018) argue that students’ perspective may have been missing because there are hardly any validated self-report instruments measuring student learning of domain-general competencies; thus, studies employing these instruments are also scarce. So far, Panadero et al. (2018) argue that the most advanced competency self-report instrument for adolescent IVET students is the one of Kyndt et al. (2014). They examined several international competency frameworks and compared these against the expectations of practitioner-level employers and vocational teachers; thus, resembling a rather occupationalistic perspective to competencies (cf. Mulder, 2014). As a result, Kyndt et al. (2014) developed a self-report instrument, which measures such domain-general competencies that may not be sufficiently addressed in the official curricula but which are highly beneficial for graduating IVET students who are about to enter the world of work, access their community of practice and encounter customers and other stakeholders. Next, we introduce the categories of this instrument.

The following four categories measure professional conduct. *Work organisation* comprises planning, organising and prioritising one’s work and usage of time (Kyndt et al., 2014). These are relatively common demands for the modern workers who are demanded to self-regulate their actions at work (Jossberger, 2010; Löfgren et al., 2020, 2022).

*Cooperation ability* incorporates teamwork and interpersonal skills. Teamwork refers to the contribution, encouragement, help and responsibility one shows in group work, while interpersonal skills include such attitudes as respect for others and politeness, which advance human interaction in general (Fox & Grams, 2007; Kyndt et al., 2014). These are important for IVET graduates because as they help others, show respect and keep up with good manners they earn the trust of their senior colleagues at the workplace and induce them to share their competence with the students (Ferm et al., 2018; Pylväs et al., 2018).

*Professional attitude* entails employee dependability (e.g., keeping up with timetables and instructions) and appropriate appearance (i.e., clothing, hygiene; Fox & Grams, 2007; Kyndt et al., 2014). In addition to these, we found in our recent studies that Finnish employers and vocational teachers expect graduating IVET students to refrain from excessive usage of mobile devices and be flexible with work tasks because these also reflect workers’ dependability and willingness to contribute to the company performance (Löfgren et al., 2020, 2022).

*Problem solving* relates to problem detection, analysis and solving (Fox & Grams, 2007; Kyndt et al., 2014). It is beneficial per se but from the employer’s point of view it signifies that an employee has developmental potential, which can result in better company performance (Kyndt et al., 2014; see also Löfgren et al., 2020, 2022). Developing employees are also resilient and willing to learn (Kyndt et al., 2014). In fact, learning competency might be the most important competency because it lays a foundation for the development of all other competencies (Mulder, 2019). As regards IVET graduates, they should be curious to learn new competencies and stay alert to recognise possibilities to learn (Jossberger et al., 2010). However, a scale measuring students’ willingness to learn was not included in the original instrument of Kyndt et al. (2014).

The remaining three categories cover competencies related to communication (Kyndt et al., 2014). *Active listening* refers to sensing and processing verbal and non-verbal information and responding to one’s communication partner respectively (Drollinger et al., 2006; Kyndt et al., 2014). *Empathy* relates to one’s sensitivity to take other people’s emotions and perspectives into account (Drollinger et al., 2006; Kyndt et al., 2014). For IVET graduates, active listening and empathy offer further means to create mutual trust and to consolidate their position in their community of practice (Ferm et al., 2018; Löfgren et al., 2020, 2022; Vähäsantanen & Hämäläinen, 2018). *Assertiveness*, in contrast, refers to the capability to stand for one’s opinion while simultaneously showing respect for others and their opinions (Kyndt et al., 2014; Martin & Anderson, 1997). IVET graduates need assertiveness to be able to question, transform and innovate their work procedures; thus, they do not just blindly comply to workplace requirements but become active agents who may better react and adapt to future changes in their profession (Mulder, 2017). In addition, assertive IVET graduates dare to stand for themselves, should they encounter inappropriate treatment (cf. Kyndt et al., 2014).

Still, a competency acquisition perspective alone may overemphasise individuals and their learning outcomes. In fact, in the Western labour market discourse youth alone have been often blamed for their lack of competencies (Atkins, 2013). Therefore, students’ experienced learning environment must be considered as well.

### Characteristics of a supportive vocational learning environment

A learning environment is a concept that incorporates access to learning space, audience, pedagogy, learning content and the anticipated learning outcomes (Moore et al. et al., 2011; Vermunt & Endedijk, 2011). It may further comprise various facilities and tools for learning, teacher- vs. student-centred approaches, collaborative learning, student activation, teacher-student interaction, school climate and other features (Forster-Heinzer, 2020; Moore et al., 2011; Vermunt & Endedijk, 2011).

Within vocational education, a learning environment is commonly conceived as a dynamic social context where students interact with their educators (e.g., teachers, workplace supervisors) and peers; thus, they gradually become active and fully acknowledged members of their community of practice (Forster-Heinzer, 2020; Jossberger et al., 2010; Lave & Wenger, 1991). A learning environment alone can help to counterbalance vocational students’ individual deficiencies and carry on with their studies, provided that educators especially pay attention to constructive and positive interaction with the students (Ryökkynen et al., 2019; Virtanen et al., 2014). Due to the social and emotional characteristics of the vocational learning environment, we concentrate on its following four global components, through which the educators may enhance student learning of competencies.

First, in a high-quality vocational learning environment educators should provide students with social support because encouragement helps students to overcome adversities and reach their potential (Forster-Heinzer, 2020; Lüthi et al., 2021; Mikkonen et al., 2017; Toom et al., 2017; Virtanen et al., 2014). Second, educators should recognise and acknowledge student endeavours for learning because this furthers student self-efficacy beliefs and professional growth (Forster-Heinzer, 2020; Toom et al., 2017; Virtanen et al., 2014; see also Lüthi et al., 2021). Third, it is highly important that the students consider themselves equally treated because this feeling of fairness highlights to students that they are judged by their accomplishments and not by their personalities; thus, their motivation is strengthened (Forster-Heinzer, 2020; Toom et al., 2017; Virtanen et al., 2014). Fourth, peer students are an essential source of joy and support in learning (Löfgren et al., 2022; Niittylahti et al., 2019; Ryökkynen et al., 2019). There should also be a constructive and positive learning climate where students may be motivated and make mistakes without peer disapproval (Böhn & Deutscher, 2021; Toom et al., 2017; see also Rintala & Nokelainen, 2020).

In Finland, vocational students study commonly both at school and in workplaces (OECD, 2020; Virtanen et al., 2014). The contemporary Finnish IVET system is a result of gradual restructuring during the past 20 years. A relatively school-based system was converted into a competence-based and workplace-learning-oriented system to improve graduates’ employability and spur the Finnish economy (Cedefop ReferNet Finland, 2019; Pylväs et al., 2018). The latest legislative reform took place between 2015 and 2018, stressed the reactivity of the IVET system to the workplace competency demands, promoted individual learning paths, further augmented the potential of workplace learning and promoted the connection of vocational schools and workplaces as a seamless learning environment (Cedefop ReferNet Finland, 2019; Pylväs et al., 2018; Rintala & Nokelainen, 2020).

Today, Finnish students access basic knowledge at school and then cultivate their competencies at apprenticeships where they may also ‘get a foot in the door’; thus, the studies between school and workplace should form a continuum (Löfgren et al., 2020, 2022; Virtanen et al., 2014). This process only succeeds when vocational teachers, workplace supervisors and other educators embrace *connectivity*: they should coordinate student guidance, negotiate learning objectives and assessment, link learning contents between different learning events and uphold a high educator ethos to care for every student (Bakker & Akkerman, 2019; Forster-Heinzer, 2020; Löfgren et al., 2022; Mikkonen et al., 2017; Ryökkynen et al., 2019; Virtanen et al., 2014). Indeed, the better the teachers and supervisors cooperate to align their practices, the better the students may profit from their multiple learning environments at school and workplaces and learn competencies (Aarkrog, 2005; Bakker & Akkerman, 2019; Böhn & Deutscher, 2021; Lüthi et al., 2021).

Scholars studying vocational learning environments have often highlighted that school and workplace are different learning environments, for example in terms of rationales, goals and cultures (Aarkrog, 2005; Rintala & Nokelainen, 2020). However, some recent studies (Lüthi et al., 2021; Mikkonen et al., 2017) also suggest that most vocational students seem to perceive vocational school and workplace as relatively aligned learning environments in terms of such situational resources as quality of instruction, educator supportiveness, climate and opportunities for learning. Also, studies from Estonia (Goodson & Ümarik, 2019), Finland (Löfgren et al., 2022; Virtanen et al., 2014) and Sweden (Nylund & Gudmundson, 2017) suggest that both technical-trade teachers and workplace supervisors tend to perceive themselves primarily as craftspersons who mediate domain-specific substance knowledge to the students and overlook their pedagogical task. The reason may be that both teachers and workplace supervisors have a background in the industry and there is a long-lasting tradition within the technical trades to socialise newcomers (i.e., students) to the prevailing conventions in the workplace community of practice (cf. Nylund & Gudmundson, 2017; Virtanen et al., 2014).

What is more, characteristic to the Finnish technical-trade vocational training is that the educators have traditionally highlighted students’ own motivation and responsibility for their studies instead of systematic educator support interwoven in the learning environment (Virtanen et al., 2014; see also Rintala & Nokelainen, 2020). Given that there is a need to further study educator-student interaction and its contribution to student development (cf. Forster-Heinzer, 2020; Ryökkynen et al., 2019), it is paramount to scrutinise this connection within technical trades in more detail.

### Aim of the study

This study aims to find out which domain-general competencies IVET students report to learn during their vocational studies. We also want to investigate how IVET students perceive the learning environment in vocational education. Moreover, we want to explore whether the experienced learning environment in vocational education contributes to student learning of domain-general competencies. Based on previous studies (e.g., Kyndt et al., 2014; Ryökkynen et al., 2019; Soini et al., 2015; Toom et al., 2017), we focus on the following research questions and define the following hypotheses:

1. How do technical-trade IVET students perceive the domain-general competencies they have learned during the studies?
  1. Students’ domain-general competencies consist of the categories of *Work organisation, Cooperation ability, Professional attitude, Problem solving, Willingness to learn, Active listening, Empathy* and *Assertiveness.*
2. How do technical-trade IVET students experience their learning environment established by vocational educators?
  H2 Students’ experienced learning environment in IVET consists of receiving *Social support* from teachers and workplace supervisors; encountering *Equality* and relatedness; having a constructive *Climate* for learning; and receiving *Recognition* for one’s opinions and efforts in learning.
3. How does the experienced learning environment relate to technical-trade IVET student learning of domain-general competencies?
  H3 A supportive, equal, constructive and encouraging learning environment contributes to student learning of domain-general competencies.

## Method

### Context

Finnish initial vocational education and training (IVET) is primarily a government-funded system. The Ministry of Education authorises all education providers and requires that they adhere to the VET curricula specified in the National Qualifications Framework (Cedefop ReferNet Finland, 2019). IVET studies cover 180 competence points and usually last three years of which students spend at least 30% and up to 80% at the workplace (OECD, 2020).

This study was executed in collaboration with four Finnish metropolitan vocational education providers. Each of these offer some 40–50 IVET study programmes and educate approximately 10,000 students annually. To allow in-depth scrutiny, the study focused on technical vocational fields. To acquire data that reflect the current trend to combine school and workplace learning environments, the study was further orientated to those programmes that had sent the most students to apprenticeships to local workplaces. Consequently, this study focused on automotive engineering, building service technology, electrical and automation engineering and mechanical and metal engineering. Each programme in each school educates approximately 600 students annually.

### Measures and data collection

The instrument of Kyndt et al. (2014) was applied in this study to collect data on student self-reported learning of domain-general competencies because it covers to a large extent the competencies that we have found to be essential for the Finnish technical IVET graduates (see, Löfgren et al., 2020, 2022). Kyndt et al. (2014) had already proved the instrument validity. Hence, the scales were adopted or omitted entirely. However, there could be minor augmentations that are explained separately. There were eight competency scales in total. In the following, if not expressed otherwise, all scales were adopted per se from Kyndt et al. (2014).

The first five scales addressed professional conduct: (1) *Work organisation* (4 items) measured respondents’ proficiency in planning, organising and prioritising. (2) *Cooperation ability* (7 items) covered teamwork and interpersonal skills. (3) *Professional attitude* (10 items) measured respondents’ dependability, appearance, appropriate behaviour and conscientious attendance. Eight of the items were developed by Kyndt et al. (2014) and comprised the original ‘professional attitude’ scale. In addition, we developed two extra items based on our previous study about employer competency expectations (Löfgren et al., 2020). These items measured respondents’ flexibility with job tasks (‘If needed, I can also perform other duties that I do not usually do’) and their excessive use of mobile devices (‘When I am in apprenticeship or at school, I mostly use my phone for working or studying’). (4) *Problem solving* (7 items) reflected respondents’ competencies in identifying, analysing and solving a problem. (5) As suggested by Kyndt et al. (2014), a new scale measuring respondents’ *Willingness to learn* (6 items) was also created (see Additional file 1). Item formulations were founded on the work of Jossberger et al. (2010); they suggest that vocational students’ willingness to learn includes curiosity and vigour in learning and a certain alertness to spot opportunities for learning.

Competency scales 6–8 (cf. Kyndt et al., 2014) dealt with communication. (6) *Active listening* (5 items) measured respondents’ proficiency in sensing and processing oral information and responding appropriately, keeping non-verbal communication also in mind. (7) *Empathy* (6 items) concerned respondents’ sensitivity to their communication partner’s emotions and ability to take another person’s perspective. (8) *Assertiveness* (4 items) measured respondents’ ability to begin and end interactions and defend one’s opinion while also respecting their conversation partner’s views and turn to speak.

The scales adopted from Kyndt et al. (2014) were translated from English into Finnish. The eight items we developed ourselves were formulated in an unequivocal and unbiased way to avoid the risk of socially desirable answers (see Braun et al., 2012). Moreover, in line with the formulations of Kyndt et al. (2014), the additional items were formulated so that participants assessed their current level of competencies rather than judging them in retrospect. Every item was rated on a five-point Likert scale (1 = ‘completely disagree’ to 5 = ‘completely agree’). In total, the 41 competency items of Kyndt et al. (2014) and eight items developed specifically for this study make a total of 49 competency items.

Besides the competency scales, the survey instrument explored participants’ self-reported experiences of their learning environment. Given that educators across different educational domains may instigate student learning by giving social support, recognising students’ endeavours for learning, treating students equally and constructing a positive learning climate (e.g., Böhn & Deutscher, 2021; Forster-Heinzer, 2020; Lüthi et al., 2021; Mikkonen et al., 2017; Niittylahti et al., 2019; Ryökkynen et al., 2019; Soini et al., 2015; Toom et al., 2017; Virtanen et al., 2014), four scales from The Student Teachers’ Sense of Professional Agency survey (see Soini et al., 2015; Toom et al., 2017) were adopted to this study due to their concise composition and already demonstrated applicability.

These scales measured the components of a supportive learning environment: *Social support* (3 items) measured the supportiveness of teachers and workplace supervisors (e.g., ‘I receive encouragement and support from teachers and workplace supervisors’); *Equality* (2 items) measured to what extent participants considered themselves equally treated (e.g., ‘I am treated equally’); *Climate* (2 items) explored how the participants experienced the learning climate among peer students (e.g., ‘I can tell openly about my failures to my peer students’) and *Recognition* (2 items) measured to what extent the participants felt themselves as accountable actors contributing to their learning environment (e.g., ‘I feel that teachers and workplace supervisors appreciate my efforts in studying’).

The scales were already validated (see Toom et al., 2017) and items were available in Finnish and English. As the scales were originally developed in a teacher education context (see Soini et al., 2015; Toom et al., 2017), minor adaptations were made to the vocabulary used. For instance, the items focusing only on teachers were reformulated to cover teachers and workplace supervisors together as this dualism reflects the similarities of technical-trade teachers and workplace supervisors in their pedagogical thinking (Goodson & Ümarik, 2019; Löfgren et al., 2022; Nylund & Gudmundson, 2017) and the current understanding of educators in the Finnish IVET (cf. Cedefop ReferNet Finland, 2019). Every item was rated on a five-point Likert scale (1 = ‘completely disagree’ to 5 = ‘completely agree’). In total, there were nine items measuring participants’ experiences of their learning environment.

The background questions were placed at the end of the survey and only necessary information was gathered to avoid stereotypical response patterns (cf. Braun et al., 2012): participants’ age, vocational school, vocational programme, study year, mother tongue (i.e., ‘Finnish’, ‘Swedish’, ‘other’) and gender (i.e., ‘male’, ‘female’, ‘other’, ‘not willing to tell’). These were needed to provide vocational schools with their own results. In total, there were six background questions. The entire instrument consisted of 64 items.

Most competency (Kyndt et al., 2014) and learning environment scales (Soini et al., 2015; Toom et al., 2017) were already validated in earlier studies. The two items measuring participants’ professional attitudes on excessive use of mobile devices and flexibility with work assignments, as well as the six items measuring willingness to learn were developed for this study by the researchers and were not validated before. However, they were based on our previous findings (see Löfgren et al., 2020, 2022) and earlier research (see Jossberger et al., 2010).

Before research material collection, the entire survey instrument went through an additional qualitative content validity check. First, item formulations were checked and approved by researcher colleagues acquainted with the field of this study. Second, the first author had a remote meeting with 15 vocational students representing the target population (see Braun et al., 2012). Students reviewed all items. They considered the items to be understandable and the length of the questionnaire acceptable. Students checking the survey did not participate in the actual data collection.

Before the data collection, research permits were obtained from all four VET providers. Study programme managers and teachers were also contacted to motivate the school staff to support the study. The actual data collection took place between November 2020 and February 2021. Due to the Covid-19 pandemic, schools provided only distance teaching and denied the access to school premises for safety reasons. In addition, the Heads of the vocational programmes stated that many students study mostly at the workplaces and seldom attend instruction at school. Students also rotate between the school and workplaces according to their own schedules; therefore, it was unlikely to contact them at school premises at once. Thus, the data were collected entirely online using Qualtrics data collection application. The participants could access the survey with their mobile devices or computers. Completing the survey took students about 15 min.

### Sample

Every student of the minimum age of 18 had the option to voluntarily take the survey. The questionnaire was presented to 1,060 upper-secondary IVET students (1,011 males and 49 females) representing four technical study programmes in four different vocational education institutions in Finland. Altogether 132 students completed the survey (see Table 1). The response rate was 12.5%. Of the total, 112 participants were males and 13 were females. Three respondents declared their gender as ‘other’ and four did not want to share. The respondents’ mean age was 22.6 years (SD = 8.87; range: 18–55 years). In comparison to the target population, the sample was relatively representative in terms of age distribution but female participants were slightly over-represented. In addition, most participants represented either School 2 or 3.

**Table 1:** Sample distribution between vocational programmes and schools

Vocational programme	School 1		School 2		School 3		School 4		**Total**	
	*N*	%	*N*	%	*N*	%	*N*	%	*N*	%
Automotive engineering	11	55.0	15	37.5	17	29.3	2	14.3	45	34.1
Mechanical and metal engineering	1	5.0	3	7.5	7	12.1	1	7.1	12	9.1
Electrical and automation engineering	8	40.0	12	30.0	20	34.5	7	50.0	47	35.6
Building service technology	0	0.0	10	25.0	14	24.1	4	28.6	28	21.2
**Total**	20	100.0	40	100.0	58	100.0	14	100.0	132	100.0

### Data analysis

To test research questions 1 and 2 and their respective hypotheses, an item-level analysis strategy would be the most precise one. Due to the relatively low sample size and high number of model parameters (cf. Rosseel, 2012) it turned out that we could not include both item-level structure and its upper, latent-level structure in the same model. Instead, preliminary bivariate correlation tests were executed to inspect whether the domain-general competencies and the components of the experienced learning environment constitute their own coherent entities (cf. Chen et al., 2005). Scale means, standard deviations and Cronbach’s alphas were also calculated for reporting purposes.

**Fig. 1:**
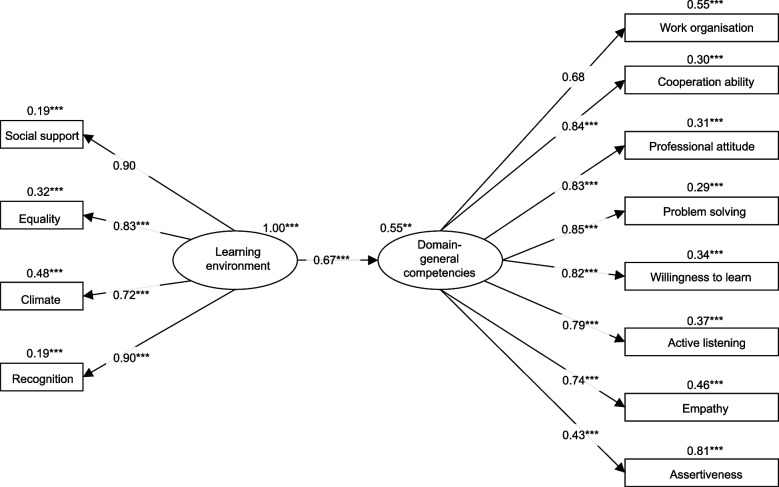
Hypothesised model of the components and relations between the vocational students’ experienced vocational learning environment (Social support, Equality, Climate, Recognition) and domain-general competencies (Work organisation, Cooperation ability, Professional attitude, Problem solving, Willingness to learn, Active listening, Empathy and Assertiveness). *Note.* Solid paths represent standardised parameter estimates (β), *p < .05, **p < .01, ***p < .001 (two-tailed)

Then, a structural equation modelling (SEM) was used to test all three research questions and their respective hypotheses in form of the hypothesised model (see Fig. 1). Instead of using the manifested variables (i.e., single items), the eight competency scales and four learning environment scales were used as composite variables (based on mean). Relations between the composite variables and latent factors were analysed with confirmatory and regression analysis strategy so that the consistency of the hypothesised model with the empirical data could be determined (Gaskin, 2021; Muthén & Muthén, 1998–2010). The analyses were conducted using the RStudio software (ver. 1.4.1717) with the lavaan package (R Development Core Team, 2021; Rosseel, 2012).

A robust MLR procedure was used to estimate model parameters and yielded maximum likelihood estimates, Huber-White standard errors and χ^2^ test statistics, which endure non-normality (Huber, 1967; Muthén & Muthén, 1998–2010; White, 1982). The goodness-of-fit of the estimated standardised model was evaluated by the χ^2^ test, Comparative Fit Index (CFI), Tucker-Lewin Index (TLI), Root Mean Square Error of Approximation (RMSEA) and Standardised Root Mean Square Error of Approximation (SRMR). A great fit with the data is indicated when the χ^2^ value is non-significant, CFI and TLI values are above 0.95, an RMSEA value is below 0.05 and SRMR value is below 0.09 (Hu & Bentler, 1999; Muthén & Muthén, 1998–2010).

## Results

### Students’ perceptions of the domain-general competencies and learning environment in the initial vocational education

To test, whether the domain-general competency domains (Hypothesis 1) and the components of the experienced learning environment (Hypothesis 2) constitute their coherent entities, we first follow the suggestions of Chen et al. (2005) and inspect the descriptive statistics (means, standard deviations) and Cronbach’s alphas for the scales (see Table 2). Alpha values exceeding 0.60 are considered acceptable while values approaching 1 indicate excellent consistency (Hair et al., 2014). In this study, most scales were sufficiently reliable (α’s = 0.57–0.90), including the new *Willingness to learn* scale (α = 0.89) developed for this study. Only the *Assertiveness* scale of Kyndt et al. (2014) had an alpha value (α = 0.57) slightly less than the generally acceptable 0.60 (cf. Hair et al., 2014). The bivariate scale correlations between the competency domains and dimensions of the learning environment were mostly statistically significant. Only the *Assertiveness* scale did not correlate statistically significantly with some learning environment scales (see Table 2).

**Table 2:** Descriptive statistics and correlations among all factors

Variable	1	2	3	4	5	6	7	8	9	10	11	12
1.Work organisation	–											
2.Cooperation ability	.65***	–										
3.Professional attitude	.51***	.68***	–									
4.Problem solving	.67***	.71***	.68***	–								
5.Willingness to learn	.50***	.67***	.71***	.72***	–							
6.Active listening	.47***	.65***	.66***	.65***	.64***	–						
7.Empathy	.44***	.61***	.67***	.56***	.52***	.71***	–					
8.Assertiveness	.33***	.33***	.38***	.42***	.23**	.41***	.38***	–				
9.Social support	.37***	.52***	.48***	.47***	.52***	.41***	.46***	.20*	–			
10.Equality	.32***	.46***	.47***	.47***	.56***	.40***	.43***	.16	.74***	–		
11.Climate	.31***	.53***	.45***	.51***	.59***	.42***	.38***	.08	.60***	.68***	–	
12.Recognition	.34***	.51***	.52***	.48***	.57***	.43***	.45***	.17	.84***	.72***	.63***	–
M	4.08	4.09	4.30	3.94	4.28	4.21	3.94	3.93	4.10	4.26	4.11	4.11
SD	.71	.67	.60	.69	.67	.66	.66	.66	.77	.87	.86	.76
α	.75	.85	.85	.90	.89	.82	.84	.57	.82	.81	.68	.72
α (Kyndt et al., 2014)	.76	.82	.88	.90		.79	.82	.72				
α (Toom et al., 2017)									.80	.81	.74	.78

^*^p < .05; **p < .01; ***p < *.001*

Students assessed their level of competencies as relatively high (Mean range: 3.93–4.30). The highest competency was *Professional attitude* (M = 4.30); thus, the students consider that they relatively diligently keep up with timetables, admit mistakes, use mobile devices primarily for work and studies at school and workplace and show flexibility with work tasks. Almost as strongly, students perceived their *Willingness to learn* (M = 4.28). They show relatively high interest towards their professional field and are curious and eager to learn. Similarly, they perceived themselves as fairly *Active listeners* (M = 4.21) who pay attention to their conversation partners and ask questions when they do not understand. Next, students considered their *Cooperation ability* (M = 4.09) quite developed; thus, they respect other people and their perspectives and are willing to compromise. Almost evenly, students perceived their *Work organisation* skills (M = 4.08) to be sufficient. They feel competent to notice if there is still work to be done, they show initiative and prioritise. Students showed caution related to their *Problem-solving* skills (M = 3.94), such as problem spotting, cause detection and modelling different solutions. They also cautiously acknowledged *Empathy* (M = 3.94) in terms of interpretation of other people’s emotions and *Assertiveness* (M = 3.93) in the instance of disagreeing constructively with others and standing up for oneself.

Furthermore, students were relatively optimistic about their vocational learning environment (Mean range: 4.26–4.10). They felt that teachers and workplace supervisors show respect towards them and treat them equally (*Equality*, M = 4.26). Students perceived the other three components of the experienced learning environment relatively evenly and positively. They felt that teachers and workplace supervisors take student opinions quite well into account and acknowledge student persistence in learning (*Recognition*, M = 4.11). Students considered that the general learning atmosphere supports learning and that peer students support each other (*Climate*, M = 4.11). Lastly, students felt that teachers and workplace supervisors support and encourage them relatively well (*Social support*, M = 4.10).

In addition, there were moderate or high correlations between the competencies (*Work organisation, Cooperation ability, Professional attitude, Problem solving, Willingness to learn, Active listening, Empathy* and *Assertiveness*, r range = 0.23–0.71) and between the components of vocational learning environment (*Social support, Equality, Climate, Recognition*, r range = 0.60–0.84). Correlations support the research hypotheses H1 and H2 and suggest that both domain-general competency domains (H1) and learning environment components (H2) should constitute their own coherent entities (Chen et al., 2005). Hypotheses H1 and H2 were tested more profoundly with the SEM model, which is presented next.

### Interrelations between vocational students’ domain-general competencies and the experienced vocational learning environment

**Fig. 2:**
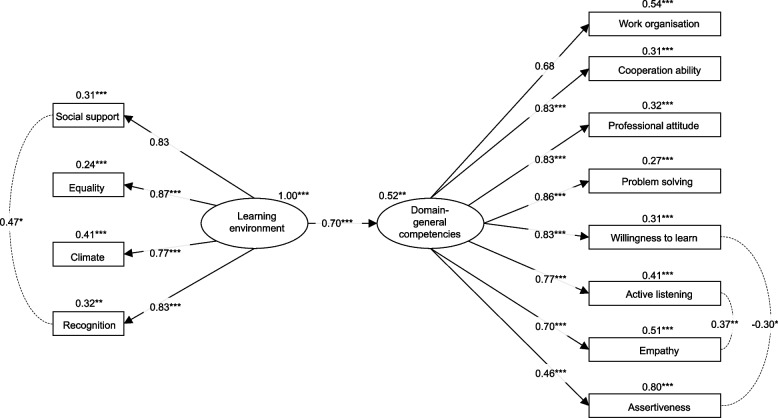
Final model of the components and relations between the vocational students’ experienced vocational learning environment (Social support, Equality, Climate, Recognition) and domain-general competencies (Work organisation, Cooperation ability, Professional attitude, Problem solving, Willingness to learn, Active listening, Empathy and Assertiveness). *Note.* Solid paths represent standardised parameter estimates (β); dotted paths are standardised residual coefficients, *p < .05, **p < .01, ***p < .001 (two-tailed)

The hypothesised model (see Fig. 1) did not yield sufficient fit with the data as indicated by the RMSEA, although the other fit indices of the standardised model were already acceptable (χ^2^(53) = 113.319, *p* < 0.001; RMSEA = 0.093 with 90% C.I. = 0.069–0.116; CFI/TLI = 0.944/0.93; SRMR = 0.056). This may be due to the relatively small dataset (*N* = 132) in comparison to the number of model parameters (cf. Rosseel, 2012). Thus, the successive, final model was adjusted with modification indices (see Fig. 2) that implied a need to add three residual correlations to the model. For that reason, an additional exploratory factor analysis (EFA) was conducted to check whether there was a need to add latent variables to the model. However, interpretation of the EFA did not provide any additional information in comparison to the SEM analysis. It is likely, that the need to add residual correlations to the model was caused by method variance, which is not important in terms of the researched phenomenon. As mentioned, the results of EFA were not reasonable; thus, they are not reported here. Next, robust maximum likelihood estimation with Huber-White standard errors was used to test the model fit (Huber, 1967; White, 1982). As a result, the robust fit indices of the final standardised model implied sufficient fit with the data (χ^2^(50) = 58.694, *p* = 0.187; RMSEA = 0.036 with 90% C.I. = 0.001–0.079; CFI/TLI = 0.99/0.99; SRMR = 0.049).

The statistically significant factor loadings in the final model (see Fig. 2) further support hypotheses H1 and H2, indicating that a factor structure is likely to exist above the components of the experienced vocational *Learning environment* and *Domain-general competencies*. In line with hypothesis 1, the vocational *Learning environment* was determined by four components: *Social support* (3 items, α = 0.82); *Equality* (2 items, α = 0.81); *Climate* (2 items, α = 0.68) and *Recognition* (2 items, α = 0.78). Corresponding to hypothesis 2, *Domain-general competencies* were determined by eight components: *Work organisation* (4 items, α = 0.75); *Cooperation ability* (7 items, α = 0.85); *Professional attitude* (10 items, α = 85); *Problem solving* (7 items, α = 0.90); *Willingness to learn* (6 items, α = 0.89); *Active listening* (5 items, α = 0.82); *Empathy* (6 items, α = 0.84) and *Assertiveness* (4 items, α = 0.57).

The final model (see Fig. 2) also gave support to the third hypothesis. Accordingly, the experienced *Learning environment* highly explained student learning of *Domain-general competencies* (R^2^ = 0.48). This result implies that the more supportive, equal, constructive and encouraging the vocational learning environment is, the more it is likely to contribute to student learning of domain-general competencies.

## Discussion

### Methodological reflections

This study commits to high research ethical standards. Researchers adhered to the individual university’s ethical guidelines. Research permits from vocational schools were applied for and granted. Schools received thorough preliminary information about the study, its aims, potential utilisation of its results and research ethical concerns. The participating students were informed similarly beforehand. They took part anonymously, voluntarily, without incentives and their consent was explicitly inquired in the online survey. The researchers presented the results in as unequivocal and unbiased a way as possible, simultaneously ensuring that participants’ anonymity remained intact.

As regards study strengths, the survey instrument was based on the already validated apparatuses examining competency self-report (Kyndt et al., 2014) and components of a supportive learning environment (Soini et al., 2015; Toom et al., 2017). Thus, this study yielded valuable information about the validity and applicability of these instruments. Moreover, this study successfully presented two new items for the *Professional attitude* scale (of Kyndt et al., 2014) and a totally new scale measuring respondents’ *Willingness to learn* as Kyndt et al. (2014) suggested. In aggregate, this study makes a methodological contribution as it examines upper-secondary IVET student perceptions to their competencies with a quantitative self-report instrument, which is a relatively scarce methodological approach in earlier studies (cf. Panadero et al., 2018).

As described in the methods section, meticulous efforts were taken to ensure study validity and reliability. Still, this study faces the following limitations. First, although the sample size (*N* = 132) was sufficient for the SEM analysis strategy applied in this study, the response rate remained relatively low (12.5%). Due to survey voluntariness, it is possible that the participants represented the most competent, independent and conscientious students. Students with motivational, reading or concentration difficulties may have skipped responding. It is also plausible that some students may have skipped answering because filling in the online questionnaire was the only possibility to take part in the survey. However, the participating schools denied access to their premises due to the Covid-19 restrictions and pointed also out a practical challenge: students seldom attend instruction simultaneously at school as many of them study mostly at the workplace (, which is characteristic for the contemporary Finnish IVET; see Rintala & Nokelainen, 2020). Thus, the researchers did not have an opportunity to contact every student face-to-face and motivate them.

Second, socially desirable survey answers commonly affect survey validity. Distorted answers can be controlled by posing clearly articulated and non-leading survey items to respondents and placing only a few necessary personal background questions at the end of the questionnaire (cf. Braun et al., 2012). These guidelines were followed in this study but the risk for socially desirable answers cannot be completely excluded.

Third, although structural equation modelling with composite variables and robust standard errors was successful in this study, the relatively small study sample (*N* = 132) did not yield a more detailed item-level analysis. Moreover, the hypothesised model had to bet adjusted with modification indices to improve model fit (cf. Gaskin, 2021). All residual correlations were statistically significant but they were irrelevant for the studied phenomenon due to EFA that showed that addition of factors did not provide a reasonable solution. Between *Social support* and *Recognition,* the residual correlation was logical as these account for the experienced learning environment. Residual correlation between *Active listening* and *Empathy* was also logical as both present social competencies. However, the residual correlation between *Willingness to learn* and *Assertiveness* raises the question of whether there were overlapping scale items or imperfect item wordings. To enable deeper item-level scrutiny, we recommend further inspection of the researched phenomenon with a larger study sample.

Lastly, the participants represented four vocational schools and four technical vocational programmes. We recommend further validation of the scales to gather information about their applicability to different contexts. Inspection would be especially beneficial for the *Assertiveness* scale. In this study, its internal consistency in terms of Cronbach’s alpha was slightly lower (α = 0.57) than what is usually expected (i.e., α = 0.60), which may decrease the scale reliability (Hair et al., 2014). It also did not turn out to correlate significantly with most components of the experienced learning environment (see Table 1). For the reasons explained above, we also note that the results should be generalised to other schools and vocational fields with caution.

### Results in light of previous literature

The results suggested that upper-secondary IVET students’ domain-general competencies can be accounted for by the competency categories of *Work organisation, Cooperation ability, Professional attitude, Problem solving, Willingness to learn, Active listening, Empathy and Assertiveness*. This finding strengthens the view of earlier, qualitative studies presenting relatively similar domain-general competency demands from vocational students or graduates (cf. Jossberger et al., 2010; Löfgren et al., 2020, 2022; Nägele & Stalder, 2017; Pylväs et al., 2018; Vähäsantanen & Hämäläinen, 2018). For another, our findings proved the competency self-report instrument of Kyndt et al. (2014) to be fairly reliable in our context. The additional *Willingness to learn* scale, which was developed specifically for this study, was also reliable and filled an existing gap in the instrument of Kyndt et al. (2014). Still, future research should examine the applicability of the instrument of Kyndt et al. (2014) and the *Willingness to learn* scale to other contexts.

Further, our findings provided a relatively rare student perspective to IVET student competency demands (cf. Billett, 2014). Interestingly, the participants of this study perceived their level of domain-general competencies to be comparatively high, while in our earlier studies employers and teachers considered students’ levels of competencies to vary or even be insufficient (Löfgren et al., 2020, 2022). Also, the students in this study gave relatively high assessments to their experienced learning environment. High-achieving students with strong self-esteem and self-efficacy beliefs tend to detect well which resources in each learning environment they may exploit to advance their learning (Lüthi et al., 2021; Mikkonen et al., 2017). Therefore, it is possible that our sample mostly consisted of high-achieving and motivated students. Future research should recruit a wider spectrum of students and collect a larger dataset to examine whether any students acknowledge their alleged lack of competencies.

Next, the results complement our understanding on how the educator behaviour relates to students’ experiences on their learning environment. As hypothesised, the students acknowledged four intertwined components of the experienced learning environment: *Social support* provided by teachers and workplace supervisors; *Equality* and relatedness, a constructive *Climate* for learning and *Recognition* of student opinions and aspirations for learning. This result coincides with the earlier findings within teacher education (Soini et al., 2015; Toom et al., 2017) and technical and healthcare vocational education (Virtanen et al., 2014), strengthens them and suggests that the same components of the experienced learning environment are important regardless of the educational context.

However, only these four social and emotional components were studied. Other relevant aspects of the experienced learning environment, such as learning facilities and contents, collaborative learning and student activation (cf. Moore et al., 2011; Vermunt & Endedijk, 2011) were not investigated in this study. Therefore, future research should go beyond technical vocational education (or healthcare; see Virtanen et al., 2014) and further investigate the components of the experienced learning environment.

Last, earlier studies have commonly addressed either student competency development or the experienced learning environment and overlooked the link between these (Forster-Heinzer, 2020; Ryökkynen et al., 2019). This study took an advanced methodological stance and employed structural equation modelling (SEM) to test the connection between the self-reported learning of domain-general competencies and the experienced learning environment. Consonant with the findings of Virtanen et al. (2014), we discovered that the experienced learning environment in IVET highly supports the learning of domain-general competencies. As we argue above, future research should examine this relation and take also other than the social and emotional components of the experienced learning environment into account.

What is more, while we investigated a unidirectional relationship between the experienced learning environment and self-reported learning of domain-general competencies, a reciprocal dynamism is also possible and worth investigating. High-achieving students seem to be capable to spot opportunities for learning in any learning environment; thus, they actively shape their learning environments to gain support and access more demanding tasks (Lüthi et al., 2021; Mikkonen et al., 2017). Therefore, future research could examine how students perceive their learning of domain-general competencies in relation to such personal resources as their perceived level of self-esteem, self-efficacy, internal locus of control and emotional stability (cf. Lüthi et al., 2021). In addition, it would be interesting to investigate to what extent the vocational educators perceive to incorporate domain-general competencies into their instruction and to compare these results to the student perceptions of their level of domain-general competencies. Our findings further yield some practical implications as explained in the following.

### Practical implications

Regarding the domain-general competencies, the student participants in our study assessed *Professional attitude* as their highest and *Willingness to learn* as their second highest competency domain. These results are positive per se but they also seemingly well coincide with the expectations that Finnish technical-trade educators traditionally pose towards their protégés: students should take responsibility of their own attitude and motivation (Virtanen et al., 2014; see also Löfgren et al., 2022; Rintala & Nokelainen, 2020). Similarly, students assessed their level of *Active listening* and *Cooperation ability* relatively high. These qualities are common requirements in the contemporary workplace-learning-oriented IVET where an ideal student, inter alia, proactively spots possibilities for learning and creates trust by cooperating for the good of the workplace community and company performance (cf. Ferm et al., 2018; Löfgren et al., 2020, 2022; Vähäsantanen & Hämäläinen, 2018). It would be interesting to investigate whether the students are as diligent, motivated and cooperating they claim to be or do they just recognise what is socially desirable to express in their technical-trade training culture (cf. Braun et al., 2012).

In fact, we find it interesting that the students gave the lowest assessments to their level of *Assertiveness*. The scale also had a relatively low internal consistency. This may imply that the student participants of our study considered the topics reflected by the scale items relatively irrelevant. Therefore, such competencies as standing for oneself and defending one’s opinion politely but firmly may not match with the traditional technical-trade training culture, which incorporates ‘master-apprentice’ hierarchies and expects that the students comply with its existing social structure and norms (e.g., Löfgren et al., 2022; Nylund & Gudmundson, 2017). Nevertheless, vocational educators should promote student assertiveness (instead of the alleged compliance) because it helps the students to think their way of working through a novel perspective; thus, they may contribute not only to their own development but also to the performance and adaptability of their workplaces (Mulder, 2017).

Next, regarding the experienced learning environment, the student participants of our study acknowledged most that their educators treat them *Equally*. Our sample consisted mostly of young male students who seek their own vocational identity and community; thus, they may have pursued acceptance from their educators and valued that they receive equal treatment in comparison to their peers (Löfgren et al., 2022; Virtanen et al., 2014; see also Niittylahti et al., 2019).

However, the slightly higher ranking of *Equality* in our data may also imply that the students in technical vocational programmes did not acknowledge enough *Social support* and *Recognition* from the educators and longed for a more constructive, positive *Climate* for learning. For example, Virtanen et al. (2014) discovered that the training culture within the technical vocational trades emphasises students’ own motivation instead of well-established learning environment. Also, Ryökkynen et al. (2019) found that Finnish vocational students, particularly in special education, rarely expect support from their educators. Moreover, we found in our earlier study (Löfgren et al., 2022) that technical vocational teachers themselves recognise that some of their colleagues do not pay enough attention to care for their students. Thus, we suggest that technical-trade educators continue to uphold student equality. In addition, they should pay attention to encounter and support students and reward their progress with encouraging feedback.

## Conclusions

This study has enriched the discussion about upper-secondary IVET students’ domain-general competencies by emphasising that a supportive, acknowledging, equal and positive learning environment highly contributes to student learning of domain-general competencies. This finding contrasts with the traditional Finnish technical-trade training culture where educators tend to emphasise students’ own motivation (Virtanen et al., 2014). While motivation and other personal resources undoubtedly help individual students to spot and exploit opportunities for learning (Lüthi et al., 2021; Mikkonen et al., 2017), our results underline that it is the learning environment that furthers student learning collectively (cf. Virtanen et al., 2014).

Vocational educators at school and also in the workplace should consider this dynamism, embrace their pedagogical task and assist their students to detect and exploit opportunities for learning. Thus, they may better safeguard that IVET students learn relevant competencies to cope with the changing world of work and altering competency requirements. Understanding this dynamism also protects students’ rights: employers, educators, politicians and other IVET stakeholders cannot blame students *alone* for lacking competencies without considering whether students had a real and fair chance to learn.

## Supplementary Information

**Additional file 1.**

## Data Availability

The dataset generated during the current study is not publicly available to protect study participant privacy as requested by the vocational institutions collaborating with the researchers of this study.
