# Supplementary material for: How does the learning environment support vocational student learning of domain-general competencies?
Source: Vocat Learn. 2023 Apr 6;16(2):343–69. doi: 10.1007/s12186-023-09318-x (PMC10078010; doi:10.1007/s12186-023-09318-x)
Supplement: Supplementary file 1 — Additional file 1. [file 12186_2023_9318_MOESM1_ESM.docx]

| **Willingness to learn scale items** | |
| --- | --- |
| 1. | I am interested in the vocational trade I am currently studying. |
| 2. | When I notice new topics and ideas in my vocational trade, I consider what I could learn from them. |
| 3. | I am eager to learn about new topics and ideas. |
| 4. | I have an open attitude to learning new topics and ideas. |
| 5. | I am curious to learn new topics and ideas. |
| 6. | At school and in the workplace, I wonder what new topics and ideas I could learn. |
| *The item scale: completely disagree 1 2 3 4 completely agree 5* | |
